# Coping strategies for existencial and spiritual suffering in Israeli patients with advanced cancer

**DOI:** 10.1186/2045-4015-3-21

**Published:** 2014-06-24

**Authors:** Netta Bentur, Daphna Yaira Stark, Shirli Resnizky, Zvi Symon

**Affiliations:** 1 Myers-JDC-Brookdale Intitute, POB 3886, Jerusalem 91037, Israel; 2 Stanley Steyer School of Health Professions, Tel Aviv University, Tel Aviv, Israel; 3 Vrei University, Amsterdam, The Netherlands; 4 Department of Radiation Oncology, Chaim Sheba Medical Center, Tel Hashomer, Ramat Gan, Israel; 5 Sackler School of Medicine, Tel Aviv University, Tel Aviv, Israel

## Abstract

Coping with existential and spiritual concerns is inescapable in end-of-life care although not enough is known about the strategies and mechanisms involved. This pilot study focused on identifying the strategies for coping with existential and spiritual suffering at the end of life of secular Jews with advanced-stage cancer. Using the phenomenological approach to data collection, in-depth interviews were conducted with 22 patients receiving symptom relief care at a daycare oncology clinic. The interviews were recorded and transcribed verbatim, and the content was analyzed.

Advanced-stage cancer patients employ several approaches to cope with existential and spiritual concerns. The themes emerging from the interviews present five dimensions of coping strategies: openness and choosing to face reality, connectedness and the significance of family, pursuit of meaning, the connection of body, mind and spirit and, lastly, humor and a positive outlook.

**Conclusions:**

Since these concerns cause suffering and distress, intervention models targeting existential and spiritual suffering should be disseminated among professionals involved in caring for people with life-threatening illnesses.

## Background

With the improvement of life-prolonging treatments, cancer patients face a more extended course of illness along with questions about mortality and the meaning of life [1-3]. Correspondingly, medical service providers and researchers have been steadily changing their orientation from focusing mainly on symptom control and physical pain management to a more holistic approach that addresses the psychological, spiritual and existential aspects of a patient's experience as tools for coping [4,5].

The issue of coping with life-threatening illness and experiences was studied more than 35 years ago by Antonovsky [6], who introduced the salutogenic theory. He argued that a sense of coherence or making sense of the world is a major factor in an individual’s management of stress and illness, and staying healthy. He explained how a sense of coherence affects health as well as the relationship between health and illness. In addition to his illness-based models, Antonovsky's [7] sense of coherence recognizes the inherent ability of the human system to counteract the tendency towards stress and disease.

There is growing understanding of the importance of resources to cope with existential and spiritual suffering at the end of life [8,9]. In addition, there is growing recognition of the significance of addressing the spiritual and existential needs of patients facing serious or life-threatening illnesses [10-12].

While there is no universal definition for existential suffering, it has been consistently understood as a complex phenomenon related to emotional distress and potentially threatening to individual integrity [13-16]. The literature offers various interpretations of spirituality, which, however, are not based on consensus [17]. They are complex, multidimensional, abstract, and ambiguous. McSherry and Cask [18] argue that linguists, social ideologists, anthropologists, researchers, and mystics are all apt to present different descriptions and interpretations of the terms.

Coping with existential and spiritual concerns is persistent and inescapable in end-of-life care, yet the underlying foundation or meaning of the concerns are not completely understood. Few studies explore the spiritual and existential concerns that dying patients are subject to. McClain et al. [19] studied the relationship between spiritual well-being, depression, and end-of-life despair among hospitalized terminal cancer patients. They found that, since spiritual well-being correlated negatively with a desire for hastened death and suicidal thoughts, it offered some protection from misery. Nelson et al. [20] studied the impact of spirituality and religiosity on depressive symptoms in patients dying from cancer or AIDS, showing that a higher score on the spiritual measure related inversely to depression. Morita et al. [21] found that hopelessness was related to existential distress among terminally-ill Japanese patients. Chochinov et al. [22] found significant correlations between the will to live and existential, psychological, social, and, to a lesser degree, physical sources of distress. In the final model, the existential variables proved to have the most influence on the will to live.

About 80% of the Israeli population are Jews; Jewish religious law pertains to everyday life [23] and the concept of "sanctity of life" (*kedushat hakhayim*) is a central value [8,14,24]. Accepting God’s ways, following traditional values and not questioning the meaning of life may make it easier for the orthodox to cope with existential and spiritual suffering at the end of life, although religiosity may or may not ultimately reduce existential suffering. However, most Jews do not live according to Jewish law even if it impacts their beliefs and culture to varying degrees. The question thus is – how do they cope with existential and spiritual concerns? Whereas religious Jews turn to God and faith, what coping strategies do secular Jews employ? This pilot study focused on identifying the coping strategies for existential and spiritual suffering at the end of life of secular Jews suffering from advanced-stage cancer.

## Methods

The study used the phenomenological approach to data collection and analysis, which seeks to describe the meaning of phenomena from the perspective of an individual's actual experience [25]. Charmaz’s constructivist approach [26] to grounded theory suggests that there is no objective, external reality for researchers to find and document, but that they must try to understand reality from the perspective of those living it. Constructivism presupposes a coproduction of knowledge by the observer and the observed, aiming at an interpretative understanding of meaning.

The main way to check phenomena in a qualitative study is through the personal experience of those living the phenomena being investigated. This study was conducted at the daycare oncology clinic at Sheba Medical Center, one of Israel’s largest hospitals. It is located in a cancer center with an in-patient ward, a chemotherapy center, a radiation therapy facility, a daycare clinic and a hospice.

The study population consisted of advanced-stage cancer patients receiving symptom relief care at the daycare oncology clinic. As is common in qualitative research, the interviewees were not selected randomly; rather, the choice was dictated by convenience and availability. The single criterion of inclusion was fluency in Hebrew or English, the singe criterion of exclusion was confusion or dementia. After obtaining approval from the medical center’s review board, the clinic’s head nurse approached the patients personally, gave them information about the study, and asked if they agreed to be interviewed. If they consented, she passed on their names to the researchers.

Ultimately, qualitative samples are drawn to reflect the purpose and aims of the study. We interviewed 20 patients and then realized that increasing the number of patients would not add to our preconceived set of categories. In keeping with the qualitative approach, we then interviewed two additional patients, which confirmed our sense that we had sufficient information and data. Fifteen interviews took place at the oncology daycare clinic, four in the oncology ward and three at patients’ homes. The average age of the patients was 58 (in a range of 39 to 76), 14 were women, 17 were married, five were single. All the patients defined themselves as secular and all lived in the Tel-Aviv area, in the center of the country. The most prevalent diagnosis was breast cancer (10 patients), followed by lung cancer (four patients), melanoma (three patients) and colon cancer (two patients). Other diagnoses were neuroblastoma, pancreatic cancer and adenocarcinoma of the stomach. Functioning and activity were measured by the ECOG Performance Status which consists of 6 grades, from 0 – fully active, to 5 – deceased. Grades 1–4 represent a gradual diminishment of physical activity, ambulatory capacity, the ability to perform activities outside and inside the house, and self-care [27]. Four patients had an ECOG Performance Status score of 3 or 4; the rest scored 2 or less. Five patients had been aware of their illness for less than one year; 12 patients were 1–5 years after diagnosis; 5 patients were more than five years after diagnosis.

All the interviews were conducted by the second author (DYS). The interviews began by inviting patients to share any thoughts or observations about their condition, and proceeded to trace the patient experiences. After this introduction, conversations took the form of semi-structured interviews. The interviewer asked leading questions – such as: "Has your illness impacted your outlook about what is important to you?" “What are your greatest concerns now?” “Are you connected to a spiritual dimension of some sort?” "How are you coping with what is happening to you?" – and invited the patients to relate to these. Then, patients were asked to reflect on the most important things at this stage of life and to describe what lent their lives meaning. Nonspecific stimuli (such as "tell me more") were used to help uncover the patients' perceptions and probe for more information. The interviews took one to two hours.

The interviews were recorded and transcribed verbatim, and the content was analyzed. The first two authors carefully read the whole transcription multiple times and, separately, assigned initial open codes to meaningful fragments of the transcriptions. The codes reflected the themes emerging from each fragment, in the language of the interviewees. The second stage consisted of structural analysis (interpretative reading) and text classification into broader categories. The first two authors made separate classifications and then discussed and unified the categories. Finally, we met several times to review and compare the findings of each interview and across all the interviews according to comparative, analytical methods.

## Results

The main focus of the transcript analysis was to extract the emerging themes and understand the patients' views of their coping strategies. Consequently, the findings are not presented numerically; we have supplied broad indications of the extent that patients expressed each theme. Herewith, we will present the interview themes that we considered most closely related to the main focus.

### Openness and choosing to face reality

Participants expressed a strong need to fully know and understand their medical condition so that they could tie up loose ends and bid their farewells. Furthermore, they had to come to terms with their imminent death. A 53-year-old woman with metastatic breast cancer said:

"I don’t need to hear everything “the nice way.” Sometimes you need to get the 10-pound hammer on the head to be in focus and accept things the way they are. I would want to know… because I need to make arrangements, to take care of things before I die, maybe make a will… yeah, not to leave loose ends. A few instructions for my husband, to have closure…"

Patients used different methods to face reality and prepare for death. A 42-year-old woman, with metastatic melanoma, said:

"I decided spontaneously to bring together all my friends from the different phases of my life; I called each and every one… I called it “a goodbye party.”

A 43-year-old woman, who had survived metastatic stomach cancer for three years, said:

*"I wrote something and gave it to a friend to keep. I said I’ll prepare a video-tape [for the children], so I prepared it all in writing and gave it to the friend… so that if something happens to me today – then they have the letter, with everything I wanted to say to them*."

However, she also expressed frustration at the reluctance of family members to talk to her about the end of life, which she herself wished to discuss:

"…..It’s important for me to talk with my kids, that they’ll know…. but we always talk about everyday things and we don’t talk about life. We pushed life aside. And that’s wrong."

At the same time, about half of the participants described a strong need to continue fighting, believing that if they would be mentally, emotionally and physically strong, they would overcome the illness. This belief functioned as a positive coping mechanism despite their awareness of the gravity of their medical condition. A woman with metastatic breast cancer said:

"Last week I heard that the doctor had given up, the illness has spread everywhere, the chemo isn’t helping, and all that’s left for me to do is start having some good times and enjoy life. That’s it. I left, and then I said ‘No! I’ll show them they’re wrong, and I’ll fight!’ [Smiling] And I am fighting.”

### Connectedness and the significance of family

Almost all the participants spoke of a need to feel connected to other people and to see others happy as an essential component of their own happiness and well-being. A 76-year-old man with metastatic lung cancer said:

"You know when I last cried? About a week ago a couple came in here. I didn’t see the man, he stood behind the curtain. The woman was handsome, tall. She held a basket. She asked, ‘Are you staying here for the Sabbath?’ - ‘Yes.’ ‘Here you go, these are sweets for all of you for the Sabbath.’ I asked her husband: ‘Sir, can you tell me what this is?’ He said ‘My son was hospitalized here for some time, he passed away. These sweets are in memoriam’. [Weeping] I started crying, that’s beautiful!”

A 39-year-old woman with metastatic breast cancer said:

"I believe in human strength, in human contact. Not as a cliché, on a true level… I think that now, around the illness, I have succeeded in reaching levels with people that are so much deeper than just the written word."

Beyond the general need for connectedness, all the participants also felt deep concern for their loved ones and placed importance on family connections. A 74-year-old man with metastatic lung cancer said:

"I had a few things that I always said I wanted to be a part of, and I achieved that, and everything is alright now… You want to see your children settled and financially secure …that their lives will be fine. I wanted a grandson, and he’s coming."

These desires, if fulfilled, may be a source of comfort and support. As a 39-year-old woman said:*"I told my boys, ‘as long as you’re happy, I will be happy… seeing you happy is my only happiness’."*

But in many cases, as their physical condition deteriorated and death became imminent, participants were distressed about being unable to continue to care for and protect their loved ones. A 60-year-old man with pancreatic cancer said:

"The thing that bothers me the most actually concerns my wife. She is most hurt by all of this. I promised myself that when she goes on vacation abroad and telephones, I’ll tell her I feel great. So that it won’t be spoiled for her."

A 69-year-old woman with metastatic breast cancer said: *"What really concerns me are my handicapped daughter, and her daughter. How will she take it, the little one [when I am gone]?"*

### Pursuit of meaning

Patients voiced a need to find meaning and purpose in their illness-related experiences. While some reflected on their lives and the illness, and gained profound insight into their relationships and goals, others saw their illness as divine retribution. The 39-year-old woman said:

"All my priorities and my perspective on life changed, I say those who haven’t been there don’t even understand what life is… I think people these days are too busy with material things and confuse the unimportant things with the essence. I’m saying there’s nothing stronger than the power of daily routine. For me, managing to keep the routine going, waking up in the morning with the kids and putting them to bed, sending them off to kindergarten with a smile, reading them a story… [is] supreme bliss. Supreme."

A 71-year-old man said:

"Scientists talk about the Big Bang, you know? So tell me, what was before the Big Bang? I want to see one scientist give me an explanation and change my mind… so yes, maybe there is a God; this is my Jewish spark. I haven’t found an answer yet. And this doubt makes me think that maybe there is something divine, which is not of the material world."

### Connecting body, mind and spirit

About half of the participants described physical and emotional states as closely intertwined and affecting one another. Examples cited were loss of human dignity, the difference between will and ability, and the need to feel good mentally in order to feel good physically. One woman said:

"If the nausea comes, I fight it. You’re not going to vomit, no, no! I hold the vomit back and it hurts in my chest to hold it back. If I’m in a good mental and emotional state, I can hold it in."

A 61-year-old woman said:

"Look I do the maximum that I can. But the body doesn’t react as I want it to or as I think it can. It’s like two parallels. I want to win and the body says “Wait a minute, Lady, I set the rules here, not you."

Another 61-old-woman said:

“I used to be as beautiful as a butterfly, now I am like… like a sack. I am sometimes embarrassed, yes… It tires me… I want, just for one night, to sew a dress together, put it on and be a butterfly again.”

### Humor and a positive outlook

Among the important coping mechanisms mentioned were a positive outlook and the ability to enjoy the small things in life. The 53-year-old woman with metastatic breast cancer said:

*"I was in a restaurant, I was eating shrimps, and I said to the shrimp “You bastard! (laughing), I am eating you now!” Really! My girlfriends laughed so hard. It was very funny. I said I want that shrimp, the one over there, I am going to eat it. And I don’t eat much of that stuff… it was difficult for me because I grew up in a religious home.*^a^*But maybe it helped me ease the burden."*

Some participants found, however, that positive coping mechanisms once effective no longer worked for them. A 44-year-old woman said:

"I used to be a person who loved laughing. We love it, my brother and I, we always got through our hard life with laughter, it’s what helped us keep our heads above water. I used humor in very difficult periods in my life… since July, when the illness came back, I just stopped laughing. I became a completely different person."

## Discussion

This study explores coping strategies for the existential and spiritual suffering of terminally ill patients in Israel. Study participants presented a total of five dimensions: openness and choosing to face reality; connectedness and the significance of family; pursuit of meaning; connecting body, mind and spirit; and humor and a positive outlook.

A comparison of our findings with other studies reveals some similar coping strategies as well as unique dimensions. Blinderman and Cherny found seven themes among the Jewish oncology population: autonomy, dignity/body image, social isolation, coping mechanisms, guilt/past disappointments, spiritual health, meaning, hope and death/dying. Based on the work of Yalon, Kissane suggested eight major forms of existential challenges: death anxiety, loss and change, freedom of choice or loss of control, dignity of the self, fundamental aloneness, the altered quality of relationships, a search for meaning, and the mystery of the unknown.

One prominent dimension similar in the three works is the need of patients to find meaning and purpose in their lives and in their illness-related experiences. We learned that while some patients reflected on their lives and the illness, and gained insight into their relationships and goals, others saw their illness as depressing and lost interest in life. Another coping strategy found in the three works, albeit with some variation, is the connection of body, mind and spirit, which is quite similar to Blinderman and Cherny's theme of dignity/body image and to Kissane's three existential challenges: loss and change, loss of control, and self-respect. Many of our interviewees described their physical and emotional states as interactive, as well as the gaps between will and ability, and the loss of human dignity. Another similarity with Kissane is the quality of relationships with family and its alteration due to illness. Indeed, most of the patients spoke of a need to feel connected to other people and to see others happy as an essential component of their own happiness and well-being. Moreover, some were deeply concerned about the future of their loved ones and their own inability to help them as, for example, the woman that worried about who would help her handicapped daughter.

As oppose to the other studies, we found that openness and choosing to face reality along with humor and a positive outlook worked for some patients and were employed as a positive coping mechanism. The different typologies, however, may stem from many reasons, such as great heterogeneity in the characteristics of the study population or the phase of their disease. In addition, since the cultural context is a significant theme in health and illness in Israel [28,29], it is important to understand it and to use the taxonomy of existential concerns of terminal patients. The cultural context may also relate to the perception of ambiguous boundaries between existential and religious domains in the field of medical care [30]. Finally, studies differ in their research methods, the analysis of researchers and their interpretations of the information [31].

For each dimension found in this study, patients developed coping strategies that helped them face the illness. At the same time, these dimensions were a potential source of suffering. For example, for many participants, one major theme was the pursuit of meaning. While in some cases this served as a supportive, effective coping mechanism in coming to terms with their medical condition, other participants were noticeably distressed as their faith was put to the test. This, despite the fact that all the patients declared themselves to be secular non-believers. According to Hermann [32], many terminal patients modified their original definitions of spirituality. Associating it at first with reference to God or various religious symbols, they subsequently modified the definition to correspond with meaning or purpose, avowing that there was room in their lives for spirituality. This is consistent with the orientation of secular societies in which spirituality is often defined as a search for meaning and all reference to a higher power is omitted, whether overtly or covertly.

Other studies also found that patients, when asked to reflect on their lives, often voice a need to discuss issues relating to the meaning of life [33,34].

Family relationships and the need for connectedness were also found to be significant dimensions in our study. This was common, too, in several other studies. McGrath [35] found that for cancer survivors and hospice patients who were still intimately involved in life through family, friends, leisure, home, and work – these were highly important in finding meaning. While most studies, including ours, elucidate the issues of connectedness and family as a single theme [8,29], the issues of family concerns, preparation and conflicted relationships may also be described separately, as was done by Morita et al. [23].

The dimensions found in this study – of openness and choosing to face reality, and of connecting body, mind and spirit – are consistent with Chochinov's insights in the article, “When Death is Near” – which is one of the most frequently downloaded articles in Canada [4]. It describes the physical changes associated with dying and the progression of change as death approaches. He argues that for many patients and families, the ability to access such information, and knowledge of the possibilities involved in response to crisis, are critical aspects of the strategy of maintaining one’s dignity.

## Conclusions

In summary, the coping strategies of Israeli patients with advanced cancer found in this study might be viewed, according to salutogenic theory, as elements of the concept of a sense of coherence. These elements are the major factor in recognizing the inherent abilities of the individual to respond to stress and disease and thus determining how well a person manages stress, illness and suffering [36].

The study has several limitations. The qualitative method does not allow for the identification of empirical causal relationships with participant characteristics. Although a variety of patients were interviewed, they do not represent Israel’s general population of terminally ill patients. The applicability of the findings to different national, social, ethnic and religious groups is therefore unknown. Furthermore, the qualitative nature of the study limits generalization. The concepts and dimensions identified may lead to the development of a larger study to explore these relationships qualitatively. In such a study, it would be of interest to receive comments from the patients about the interview itself and examine whether the fact of having someone listen to them is likely to have a therapeutic impact.

### Implications for policy

While some medical professionals have started to recognize the spiritual and existential aspects of suffering, they have only a vague perception of their meaning and importance, and this obviously limits their ability to address them. Attempts should be made to expand the number of professionals who are exposed to the notion of existential suffering, and to deepen their understanding of its dimensions. Consideration should be given to the idea of providing psychologists and social workers interested in working with terminally ill patients with specialized training that emphasizes issues related to existential suffering. This training would include special interventions that afford patients an opportunity for greater comfort at the end of life. Such intervention models have been developed in other countries in the past decade, specifically targeting these issues in patients with life-threatening illnesses [13,30,37]. Most are group interventions while a few are individual, mainly short-term treatments. Naturally, these programs would need to be adapted to the needs of patients in Israel, and to the structure and procedures of the local health services so that they could be introduced and incorporated in a system with limited resources. In addition, these interventions should be manifest and disseminated among both patients and professionals involved in end-of-life care. Concomitantly, professionals should be educated to increase patient referral to interventions designed to improve the quality and conditions of the end of life and dying [4].

An example of such program may be the Dignity Therapy, which was originally designed to reduce spiritual or existential distress in terminally ill patients [22]. Trained dignity therapists guide patients through discussion, touching on matters of the utmost importance to them or that they would most want others to remember following their death. The therapist follows the respondent's cues, facilitating the disclosure of thoughts, feelings, and memories. The therapist follows the Patient Dignity Inventory (PDI) which is a novel 25-item psychometric instrument that provides some structure but is highly flexible in order to accommodate individual needs and choices. After transcribing the conversation, the transcription is returned to the patient. An intervention of this type, that is based on an empirically grounded model of dignity, and has been proven [22,38] to be effective in reducing end-of-life existential distress, could be culturally adapted to key Israeli sub-populations.

To encourage adoption of such innovative approaches, policymakers might consider providing special funding for pilot projects and their evaluation.

## End note

^a^The word ‘cancer’ in Hebrew also means a type of crab, which is not eaten according to Jewish dietary laws.

## Competing interests

The authors declare that they have no competing interests.

## Authors' contribution

DYS, NB and ZS designed the study, DYS carried out the interviews, DYS and NB analazyed the interviews, SR reanalyzed the interviews, NB and DYS drafted the manuscript, SR and ZS gave comments and NB wrote the final manuscript. All authors read and approved the final manuscript.
